# The fat‐heart entanglement and the role of ‘osteopontin mechanics’ in cardiometabolic senescence

**DOI:** 10.1111/eci.70181

**Published:** 2026-01-21

**Authors:** Cristina Michelauz, Fabrizio Montecucco, Luca Liberale, Federico Carbone

**Affiliations:** ^1^ First Clinic of Internal Medicine, Department of Internal Medicine University of Genoa Genoa Italy; ^2^ IRCCS Ospedale Policlinico San Martino Genoa – Italian Cardiovascular Network Genoa Italy

**Keywords:** aging, atherosclerosis, heart failure, obesity, osteopontin, senescence, visceral adipose tissue

## Abstract

**Background:**

Residual cardiovascular (CV) risk persists despite therapeutic advances. Obesity is heterogeneous, and visceral adipose tissue (VAT) dysfunction (‘adiposopathy’) complicates risk stratification. Osteopontin (OPN) is a pleiotropic mediator implicated in VAT inflammation, senescence‐associated pathways, atherosclerosis and myocardial remodelling.

**Methods:**

We performed a narrative synthesis of preclinical and clinical evidence on OPN across three domains: (i) VAT biology and senescence‐associated secretory phenotype (SASP); (ii) atherosclerosis and plaque vulnerability; and (iii) myocardial fibrosis/remodelling and cardiometabolic heart failure. We also summarize biomarker and therapeutic implications.

**Results:**

OPN is increased in obesity, particularly within VAT, where it contributes to SASP‐related signalling, macrophage dysfunction, low‐grade systemic inflammation, insulin resistance and cardiometabolic risk. Clinical and translational studies link circulating and tissue OPN levels to vascular inflammation, plaque vulnerability and adverse CV events. In the myocardium, OPN appears to act as a remodelling‐specific mediator connecting extracardiac adiposity‐related signals to interstitial fibrosis, with the strongest evidence in heart failure with preserved ejection fraction and diabetic cardiomyopathy. Across settings, OPN shows promise as an early biomarker for detection and risk stratification. Experimental modulation of OPN attenuates fibrosis and improves cardiac function in multiple models, supporting its candidacy as a mechanistic therapeutic target.

**Conclusions:**

OPN likely bridges the fat–heart axis through senescence‐related pathways and may help explain residual CV risk in obesity‐related cardiometabolic disease. Standardized assays, prospective validation and interventional studies are required to establish clinical utility for OPN as a biomarker and therapeutic target.

## Graphical abstract

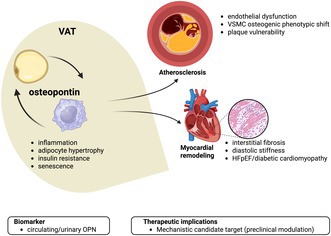

## 1 INTRODUCTION

The paradigm of cardiovascular (CV) risk has been in continuous evolution for two decades. While advances in pathophysiology and pharmacotherapy have led to a significant reduction in the worldwide burden of CV risk, new challenges are still emerging. The ‘lipid story’ has taught us that even optimal treatment leaves most patients still at risk for recurrent events. From a post‐hoc analysis of the FOURIER trial to contemporary ones, the concept of residual CV risk is globally stressed as an urgent unmet clinical need.^1^, ^2^ Lowering of high‐sensitivity C‐reactive protein would deal with residual CV risk but it seems only the tip of the iceberg. The paradigm of metabolic syndrome (MetS) as conceived by Prof. Reaven in 1988 is being challenged, but it still provides the lens to frame the role of inflammation in residual CV risk.^3^ The term ‘adiposopathy’ well clusters some central tenets of residual CV risk: the shift toward visceral and ectopic fat deposition, insulin resistance and pro‐inflammatory phenotype.^4^ Recent perspectives on adiposopathy are framing it into the broader paradigm of aging.^5^, ^6^ Driven by the complex heterogeneity and an ever‐growing prevalence worldwide, current research field of obesity is focusing on the upstream pathways related to the aging process.^7^ In a provocative fashion, the impact of obesity science is already reverting onco‐cardiology *like the shift from Newtonian to relativistic physics* ^8^ and has potential to upset the foundations of current CV knowledge and treatment.^9^, ^10^, ^11^ This narrative review focuses on the still elusive fat‐heart entanglement. We borrowed this term from quantum physics where the term quantum entanglement refers to:

generated particles that interact in a way such that the quantum state of each particle cannot be described independently of that of the others, even when they are separated by a large distance.

$$\left(i\partial \;/-m\right)\;\psi =0.$$

Our goal is to summarize the role of osteopontin (OPN), a sialoprotein with a broad distribution and range of biological activities. In recent years, OPN has been proposed as an active player in age‐related adipose tissue remodelling and enhancer of CV risk.^12^, ^13^ We then discuss the current knowledge in OPN biology and its role in cardio‐metabolic disease including any potential for translation in clinical practice.

## 2 OBESITY DOES NOT BEHAVE LIKE A FIXED BUT RATHER A FLEXIBLE MULTI‐DIMENSIONAL SYSTEM

Obesity is a heterogenous and dynamic condition, which hardly fits a consensus definition. While stakeholders worldwide still get stuck in the recommendation of body mass index (BMI) alone for definition and risk stratification, this measure is inherently limited and harbinger of paradoxes. The full strength of the association between BMI and CV risk is indeed not fully realized until adjusted for waist circumference.^14^ Like a reloaded version of the ‘Vitruvian man’, the last decade witnessed a plethora of anthropometric measures with the potential for refining obesity phenotyping. However, whether their combination can improve the obesity phenotyping and identify any excess cardio‐metabolic risk remains uncertain. Nevertheless, the meaning of defining obesity gains full significance when associated to the related cardio‐metabolic risk. Meta‐analyses testing the predictive role of BMI alone have yielded inconclusive results or even reported a U‐shape correlation with prevalent or incident cardio‐metabolic risk.^15^ The right part of the ‘U’ curve – also referred to as ‘obesity paradox’ – has been explained by an arbitrarily defined obesity phenotype known as ‘metabolically healthy obesity’ (MHO).^16^ MHO usually refers to individuals with obesity and two or fewer defining criteria for metabolic syndrome, while approximately 10% of them have none of the criteria, and as few as 5% have normal insulin sensitivity.^17^ Furthermore, MHO carries a substantially higher cardio‐metabolic risk compared to lean individuals, along with a high rate of transition to unhealthy state.^18^ Therefore, any crude categorization across categories still leads to the loss of important information dealing with qualitative and temporal patterns.

Beyond the mass of adipose tissue, the metabolic heterogeneity of obesity is closely tied to the concept of ‘adiposopathy’, firstly theorized in a seminal paper dating back to 2018.^4^ Three central tenets of ‘adiposopathy’ were identified: shift toward visceral and ectopic fat deposition, insulin resistance and pro‐inflammatory cytokine dysregulation. The hypertrophic growth of adipocytes is likely the trigger of a pathological phenotype characterized by hypoxia, fibrosis and leukocyte infiltration. Functionally, adipocyte overload and failure – defined as an impaired capacity to store the excess energy from free fatty acids (FFA) – would lead to a ‘spillover’ of excess fat to other tissues, insulin resistance and a systemic state of low‐grade chronic inflammation.^19^ Macrophages are central to adipose failure. Their number and encoded proteins correlate with adipocyte size, body mass and insulin sensitivity.^20^, ^21^, ^22^ Obesity creates a microenvironment where different factors orchestrate the pro‐inflammatory polarization of adipose tissue macrophages (ATM): hypoxia, hyperinsulinemia/hyperglycemia, FFA. The development of single‐cell technologies is deepening insight into the high heterogeneity and plasticity of ATM. Beyond the classical pro‐inflammatory M1, ATM differentiate into a plethora of different metabolically active phenotypes: lipid‐associated macrophages, regulatory macrophages, perivascular‐ and non‐perivascular‐like macrophage, collagen expressing macrophages and proliferating lipid‐associated macrophages.^23^, ^24^ Similarly, while cytokines ignite the crosstalk between adipocytes and ATM to build up a link with insulin resistance and systemic inflammation, dramatic progress has been made in understanding the complex signalling network between those cells.^25^, ^26^ The role of microRNAs delivered through exosomes or microvesicles is hypothesized. Evidence is accumulating on how adipocyte‐derived miRNAs can modulate ATM phenotype,^27^, ^28^ whereas both ATM‐ and adipocyte‐miRNAs have been shown to modulate insulin sensitivity in vivo and in vitro.^29^, ^30^, ^31^ Even more recently, the functional mitochondria transfer has been identified as further adipocyte‐to‐macrophage axis, which is suppressed in obesity.^32^

More generally, the contemporary view of obesity is shifting focus toward aging as a potential ‘theory of everything’ linking ‘adiposopathy’, insulin resistance and immune(senescence) patterns. There is substantial evidence that obesity and insulin resistance are related to cellular senescence, as expressed by a state of stable growth arrest caused by DNA damage, telomere attrition, metabolic dysregulation and organelle stress.^33^ As with aging, obesity/insulin resistance lead both adipocytes and the immune system toward a dysregulated response characterized by a defined spectrum of released factors referred to as senescence‐associated secretory phenotype (SASP).^7^ Since reducing SASP burden is likely a mechanism by which senescent cell elimination improves aging conditions, there is great interest in human translation. OPN is increasingly reported as a marker of senescence, associated with frailty and medical risk in aging.^5^, ^34^ The following paragraphs will focus on mechanistic insights and clinical relevance of OPN in fat‐heart entanglement.

## 3 OSTEOPONTIN AND VISCERAL ADIPOSE TISSUE: LIKE A DOUBLE‐SLIT EXPERIMENT

While OPN is historically known as an active player of bone remodelling with deep gene regulation in osteoblasts and osteoclasts, it actually exerts pleiotropic activities. The binding of integrins and CD44 accounts for most of the biological effects of OPN, once cleaved in some of its different cleavage sites. Many of these effects are related to the immune system, ranging from innate to adaptive response and from acute to chronic phases.^35^ OPN is highly secreted by macrophages and orchestrates their migration and phagocytic properties, and it is also critical for NK expansion,^36^ dendritic cell generation/differentiation and neutrophil function.^37^, ^38^ Beyond this, OPN modulates the entire T cell function from generation to maturation, polarization and activation.^39^, ^40^, ^41^ In late stages, OPN is essential for proper wound healing. However, while OPN deletion leads to a less organized matrix deposition (i.e. with reduced number and diameter of collagen fibres), a persistent excess of OPN leads to fibrosis.^42^

In the last decade, OPN has emerged as a potential bridge between the central tenets of adiposopathy. Noteworthy, the role of OPN has been highlighted for both dysmetabolic and pro‐inflammatory effects. Such a paradoxical role recalls the double‐slit experiment, a milestone of modern physics demonstrating that:

light and matter can satisfy the – seemingly – incongruous classical definitions for both waves and particles.

While this ambiguity fueled the fundamentally probabilistic nature of quantum mechanics, an ever‐deeper knowledge on the role of OPN might potentially improve the knowledge on the meaning of the ‘cardio‐metabolic’ term till the ‘reverse cardio‐oncology’ paradigm.

Early in 2007, serum levels of OPN were first observed in people living with obesity, where the expression of mRNA was greater within omental adipose tissue. A decrease in serum levels also occurs after weight loss.^43^ Due to substantial receptor expression in both immune cells and adipocytes, OPN deeply interferes with metabolic function. While OPN sustains a pro‐inflammatory microenvironment through the release of cytokines, the concentration‐dependent inhibition of both peroxisome proliferator‐activated and adiponectin receptors expression suggests a direct interference of OPN on insulin resistance.^44^, ^45^, ^46^, ^47^, ^48^ Noteworthy, OPN deletion in high‐fat feed mice improves insulin sensitivity despite any effect on body weight, composition or energy expenditure.^49^

A seminal paper published in 2021 in Nature Medicine finally brought many hallmarks of obesity, identified over a decade ago, under the umbrella of senescence.^7^, ^50^ Hypertrophic adipocytes enter the cell cycle in obesity, while hyperinsulinemia is correlated with adipocyte senescence. Without any evidence of a mitotic fate, the enrichment of G2 transcript – especially of cyclin D2 – is associated with transcriptional pathways of cellular senescence and a coherent secretome referred to above as SASP.^7^ OPN belongs to the SASP released by senescent hypertrophic adipocytes^51^, ^52^ and ATM. ATMs acquire a p16‐associated senescence‐like phenotype in obesity, where OPN is one of the most discriminative upregulated genes.^53^ OPN deletion and transplantation of OPN^−/−^ bone marrow‐derived macrophages in mice reveal its functional link with the senescence‐like ATM phenotype, preserving/restoring a balanced M2 polarization and critical functions like phagocytosis and efferocytosis. Even more, while the senescent‐like ATMs propagate dysfunction to the neighbouring VAT cells, OPN^−/−^ bone marrow graft rejuvenates VAT and restores its metabolic function.^53^

## 4 OSTEOPONTIN IN THE ENTANGLEMENT BETWEEN VISCERAL FAT AND HEART DISEASE

Although the role of biological age is upfront in cardiometabolic risk assessment and a target of many algorithms, the effect of accelerated aging – and the potential for slowing/reverting it – is largely unknown.^54^ Aging seems now, to some degree, modifiable, allowing chronological and biological age to be uncoupled. It is even argued – in a visionary fashion – that aging is a *modifiable* risk factor for cardio‐metabolic diseases.^55^, ^56^, ^57^

While *age* is a documented risk factor for CV disease, classically included in risk scoring systems, *aging* has a stronger residual effect that extends beyond the longer exposure to classical risk factors. The low estimated contribution of age to cellular *senescence* (11.9% and 40.3% for men and women, respectively) suggests a substantial void covered by other in parallel and likely intertwined processes.^58^ Whether what is commonly defined as ‘residual CV risk’ is attributable to premature or accelerated senescence is a fascinating, albeit still speculative, hypothesis.^10^ However, it finds support in other contexts, such as laminopathies (e.g. Hutchinson‐Gilford progeria and Werner syndrome) and cancer therapies.^59^, ^60^ In this view, the VAT‐generated SASP would sustain a fat‐heart entanglement triggering distant non‐senescent cells to undergo ‘secondary’ senescence or accelerating this process where locally underway.^61^

### 4.1 Atherosclerosis

There is a growing consensus on the detrimental effect of biological aging in atherosclerosis, where foamy‐like ECs, vascular smooth muscle cells (VSMCs), macrophages and T cells all exhibit a senescent pattern from the early stage of disease.^62^, ^63^ Endothelial cell senescence directly compromises barrier integrity in response to oxidant/senescent stressors and promotes further senescence.^64^ Conversely, VSMCs acquire an osteoblastic secretory phenotype – including OPN release – sustaining necrotic core enlargement and plaque calcification.^65^, ^66^ Remarkably, the removal of p16Ink4a^+^ foamy macrophages even leads to significant lesion regression and improvement in plaque stability.^67^, ^68^

In this context, the involvement of OPN in the atherosclerotic disease has been widely reported across the last decade (Table 1).^69^, ^70^, ^71^, ^72^, ^73^, ^74^, ^75^

**TABLE 1:** Summary of studies describing the association between osteopontin and atherosclerotic disease.

Authors	Year	Study type	Patient characteristics	Outcome(s)	Results
Sponder et al.^69^	2016	Cross‐sectional	223 non‐or ex‐smoking CAD patients	MAC	OPN significantly increases with MAC. The observed increase in OPN is dependent on MAC severity: from having no MAC (102.9 ng/mL) to mild and moderate/severe stage (122.7 and 187 ng/mL). This corresponds to an increase of 83.3% (*χ* ^2^ = 20.3; *p* < .001). OPN significantly differs between no MAC and each MAC group (*p* = .023; *p* = .001), but not between the two MAC groups (*p* = .135). At logit OPN is independently associated with the likelihood of MAC (OR 1.007; *p* = .006) and correctly classified 56.8% of cases.
Carbone et al.^70^	2019	Case–control	80 SLE and 80 matched controls	cIMT	Besides significant differences in CV risk factors (i.e. waist circumference, hypertension and dyslipidemia) and the inflammatory status between groups, OPN was positively associated with mean cIMT (*r* = .364; *p* = .001) in SLE patients also in multivariate analysis (*β*_adj_ = .240; *p* = .027).
Wirestam et al.^71^	2021	Case–control	60 SLE and 60 matched controls	cIMT	Higher plasma levels of OPN are found in SLE group and further increase with concomitant APS (*p* = .03 and .004, respectively). However, there was no significant correlation between OPN and cIMT.
Behairy et al.^72^	2021	Cross‐sectional	80 chronic HD patients	cIMT	The upper OPN tertile (78–270 ng/dL) has the highest incidence of atherosclerosis being significantly correlated with cIMT (*r* = .533, *p* = .001). Performance of OPN in detecting atherosclerosis is characterized by 70% sensitivity and 69% specificity of 69%. The AUC is .769.
Carbone et al.^73^	2022	Cross‐sectional	544 subjects from CAPIRE study categorized as low (*n* = 322) and high (*n* = 222) CV risk factor burden	CAD	The highest OPN levels are found in the low risk factor group, where they are associated with CAD (23.2 vs. 19.4 ng/mL; *p* = .001). Limited to this group OPN is independently associated with CAD (OR_adj_ 8.42 [95% CI 8.42–46.83]; *p* = .015).
Maddaloni et al.^74^	2023	Cross‐sectional	848 DM patients enrolled in the SUMMER study	History of CVD DR	OPN doesn't differ in patients with or without history of CVD but is higher in those with DR (median 3.1 vs. 2.8 ng/mL).
Balmos et al.^75^	2023	Cross‐sectional	119 patients undergoing CEA	cIMT	Intraplaque OPN expression is higher and tightly associated with the presence/extent of the inflammatory infiltrate. Similarly, OPN was higher in ulcerated plaques (*p* = .022).

*Note*: Pubmed search: ((osteopontin [Ti]) OR (OPN [Ti])) and ((atheroscler* [Ti]) OR (plaque [Ti]) OR (cardio* [Ti])).

Abbreviations: APS, antiphospholipid syndrome; AUC, area under the curve; CAD, coronary artery disease; CI, confidence interval; cIMT, carotid intima media thickness; CV, cardiovascular; CVD, cardiovascular disease; DR, diabetic retinopathy; MAC, mitral annulus calcification; OPN, osteopontin; OR, odds ratio; SLE, systemic lupus erythematosus.

First investigated in vascular calcification processes,^69^ OPN has over time broadened its relevance in atherosclerotic disease. At least two studies analysed the relationship between OPN expression – both systemically and locally – with specific plaque features. OPN indeed acts as critical pro‐inflammatory molecule promoting recruitment and activation of innate immune cells.^12^, ^38^, ^75^ These histological findings have clinical relevance as directly associated with atherosclerotic plaque ulceration and related atherothrombotic events. Paradoxically more nuanced is instead the cross‐sectional of OPN and any radiological evidence of atherosclerotic disease presence and extent. Despite limited to small sample cohorts, mostly biased by co‐existing inflammatory diseases (e.g. systemic lupus erythematosus),^70^, ^71^ the effect of OPN seems quite small or rather suggests a sub‐optimal framework. The CAPIRE study has taught a lot about the need to correctly re‐frame the traditional concept of patients' vulnerability and how OPN should fall within.^73^, ^76^ Specifically designed to study outlier patients, the presence of coronary artery disease (CAD) interacts with different burden of CV risk factors (low/high).^77^ In this study, OPN is higher in the low risk factor group, with the highest levels observed in patients with CAD. Ultimately, the insights from CAPIRE study allow to substantially speculates that: (i) the persistently high prevalence of CAD is attributable to residual CV risk and (ii) OPN may well summarize the hallmarks of residual CV risk, which can be understood in the context of cardiovascular aging.^10^

### 4.2 Myocardial remodelling

The higher prevalence of heart failure (HF) during later years of life is mainly attributed to the preserved ejection fraction (HFpEF) phenotype. In adults older than 90 years, HFpEF is almost the exclusive cause of HF and is associated with high comorbidity burden, higher hospitalization/mortality rate and ultimately poorer quality of life than the reduced ejection fraction phenotype.^78^, ^79^ Diastolic stiffness acts as a substrate for HFpEF, with structural changes ranging from cardiomyocyte decrease and enlargement to increased non‐myocyte matrix deposition. As those changes are age‐related and attributed to the accumulation of senescent cell,^80^ ‘weighty’ matters are raised for HFpEF. The influence of body fat/lean composition on HFpEF is indeed not limited to the cardiorespiratory fitness^81^, ^82^, ^83^, ^84^, ^85^, ^86^, ^87^, ^88^ but would drive an accelerated myocardial senescence. Substantial evidence associates HFpEF with ectopic fat depot in the liver^89^, ^90^, ^91^, ^92^, ^93^, ^94^, ^95^, ^96^, ^97^ but especially at the epicardial layer.^98^, ^99^, ^100^, ^101^, ^102^, ^103^, ^104^, ^105^, ^106^ In this view, it has been speculated on HFpEF as a multisystemic syndrome with prominent extracardiac involvement and different phenotypes. Considering the main comorbidities for HFpEF – metabolic disturbances/obesity and aging – their clustering would elicit the most prevalent HFpEF phenotype also referred to as ‘cardiometabolic HFpEF’.^107^

An additional related concept is the ‘functional obesity paradox’ recently described at the level of the coronary circulation, where a U‐shaped association between BMI and hyperemic myocardial blood flow has been demonstrated in a PET‐based study of coronary circulatory function.^108^ This pattern – characterized by progressive impairment from normal weight to obesity followed by paradoxical normalization in morbid obesity – likely reflects the divergent cardiometabolic effects of visceral versus subcutaneous adiposity, further reinforcing that BMI alone masks profound heterogeneity in adipose biology. Notably, the endocannabinoid system links obesity, coronary microvascular dysfunction and cardiometabolic senescence. Circulating anandamide and 2‐arachidonoylglycerol are strongly associated with impaired endothelium‐dependent and hyperaemic myocardial blood flow in individuals with obesity, indicating early microvascular dysfunction driven by adipose‐derived mediators.^109^ Consistently, gastric bypass–induced weight loss normalizes coronary circulatory function in parallel with reductions in anandamide and increases in adiponectin.^110^ Endocannabinoid signalling promotes oxidative stress, macrophage recruitment and low‐grade inflammation—processes that converge mechanistically with OPN‐mediated VAT–heart remodelling and help frame coronary microvascular dysfunction as an early manifestation of cardiometabolic senescence. Although OPN was not assessed in those cohorts, these observations are mechanistically consistent with our framework: OPN closely mirrors VAT inflammation, macrophage senescence and SASP burden, features enriched in metabolically unhealthy obesity and potentially linked to the decline in coronary microvascular reserve observed in intermediate BMI ranges.

OPN has long been labelled as a ‘remodelling‐specific biomarker’^111^, ^112^ with a detailed pro‐fibrotic effect^113^, ^114^, ^115^, ^116^, ^117^, ^118^ encompassing different phenotypes of diastolic dysfunction. Beyond HFpEF,^119^, ^120^, ^121^ OPN has recently gained high relevance for diabetic cardiomyopathy. While OPN deletion alone reduces fibrosis and improves myocardial function in streptozotocin‐induced diabetic mice,^122^ we have recently brought out a diagnostic role for OPN in the very early stage of diabetic cardiomyopathy, when microalbuminuria is not established yet. Among the most updated determinants – left ventricular (LV) diastolic dysfunction (LVDD), LV hypertrophy and left atrial enlargement (LAE) – OPN was found to be independently associated with LVDD and the prevalence of diabetic cardiomyopathy whether defined by both ≥1 or ≥2 criteria.^123^

The pathophysiology of diabetic cardiomyopathy raised additional ‘weighty’ matters^124^ about the source of OPN and the body fat/lean composition. OPN expression is not constitutive of healthy myocardium but locally triggered by mechanical stress (e.g. pressure overload, hypoxia and cardiac injury). Finally, a seminal paper published in 2018 defined the critical role of VAT in the progression of interstitial myocardial fibrosis through the production/release of OPN.^125^ By observing 2‐ and 14‐month‐old mice, OPN significantly increased during aging, with VAT showing the strongest OPN induction, contrasting with myocardium that did not express OPN. Both OPN deletion and treatment with the small‐molecule OPN inhibitor agelastatin A fully reversed age‐related myocardial fibrosis and dysfunction. This evidence ultimately represents a milestone of fat‐heart entanglement and strongly supports the bridging role of OPN.

While we previously reviewed the perils of epicardial fat on atrial failure,^126^ a recent single‐cell transcriptomic analysis of human atria has documented the expansion of SPP1^+^ macrophage as a promoter of atrial fibrillation.^127^ Macrophage‐derived SPP1 is a pleiotropic AF catalyst in HOMER mice (i.e. combining hypertension, obesity and mitral valve regurgitation) so that bone marrow transplantation from Spp1^−/−^ to HOMER mice prevents left atrial enlargement (LAE), LV hypertrophy and ultimately AF inducibility and burden. Not surprisingly now, SPP1 encodes the matricellular protein OPN.

Accordingly, current evidence indicates that targeting the cardiometabolic substrate of HFpEF is more feasible than directly aiming at OPN levels. Recent consensus statements by the European Society for Clinical Investigation emphasize that obesity‐related HFpEF should be approached as a distinct cardiometabolic phenotype in which visceral and epicardial adiposity, systemic inflammation and ectopic fat depots represent actionable targets beyond BMI.^128^ In line with this view, incretin‐based therapies such as tirzepatide have been shown to reduce cardiovascular death or worsening HF events, improve health status and functional capacity and decrease left ventricular mass and paracardiac fat in patients with HFpEF and obesity, with comparable benefits in individuals with and without type 2 diabetes despite a smaller absolute weight loss.^129^ These findings suggest that drugs capable of shrinking visceral/epicardial fat and attenuating low‐grade inflammation may indirectly modulate OPN‐driven myocardial senescence and fibrosis, even though OPN itself is not yet a formal treatment target but rather a promising biomarker to identify high‐risk HFpEF phenotypes.

## 5 CLINICAL EXPERIENCE ABOUT THE USE OF OSTEOPONTIN IN CARDIO‐METABOLIC RISK STRATIFICATION

The use of OPN as a single biomarker has not yet been validated for cardio‐metabolic risk stratification. It has been occasionally reported in different settings, especially when inflammatory diseases co‐exist (see Table 1 above). A substantial but not unanimous body of literature associates OPN with CV risk in the context of chronic kidney disease,^72^, ^130^, ^131^, ^132^, ^133^, ^134^, ^135^, ^136^, ^137^, ^138^, ^139^ alone or in multi‐panel assays.^138^, ^140^ Notably, the classical OPN assay on serum is now complemented by urine measurement, especially in non‐albuminuric diabetic kidney disease.^48^, ^141^, ^142^

More generally, higher serum levels of OPN traditionally associate with vascular degeneration^143^ and atherosclerotic disease, from subclinical stage (see Table 1 above) to late adverse events (Table 2).^12^, ^13^, ^140^, ^143^, ^144^, ^145^, ^146^, ^147^, ^148^, ^149^

**TABLE 2:** Summary of longitudinal studies analysing the predictive value of osteopontin toward adverse cardiovascular events.

Authors	Year	Patient characteristics	Outcome	Results
Abdalrhim et al.^144^	2016	3567 CAD patients from PEACE trial 4.8y median FU	CV death Non‐fatal MI HF hospitalization	LnOPN significantly associates with CV death risk (HR_adj_ of 1.24 [95% CI 1.01–1.52]; *p* = .04) and incident hospitalization for heart failure (HR_adj_ 2.04 (95% CI 1.44–2.89); *p* < .001).
Arsenault et al.^145^	2016	1.424 CAD subjects from TNT study with or (*n* = 253) or without DM with late incidence or not (*n* = 101/1070) 4.9y FU	Incident DM	Plasma lipids, adiponectin and Lp‐PLA2 but not OPN significantly predict late incidence of DM during FU in statin‐treated CAD patients.
Feldreich et al.^146^	2017	741 subjects form the ULSAM cohort 8y FU	Incident CKD CV death risk	Higher uOPN, but not pOPN, is independently associated with incident CKD (OR_adj_ SD increase 1.42 [95% CI 1.00–2.02]; *p* = .048). Conversely, pOPN, but not uOPN, is independently associated with CV death (HR_adj_ per SD increase 1.35 [95% CI 1.14–1.58]; *p* < .00).
Tang et al.^143^	2018	15,792 subjects from the ARIC study 24y FU	AAA	MMP‐9 and IL‐6, but not OPN, are associated with risk of developing AAA, independently of established risk factors.
Carbone et al.^12^	2018	225 patients undergoing CEA asymptomatic (*n* = 185) or symptomatic (*n* = 40) for IS 24‐month FU	ACS IS MACEs	OPN is 2‐fold higher in symptomatic patients. Serum OPN values >70 ng/mL confer a significant increased risk of ACS (HR_adj_ 8.28 [95% CI 1.70–40.46]; *p* = .009), IS (HR_adj_ 12.52 [95% CI 1.40–111.93]; *p* = .024) and the composite outcome MACEs (HR 13.14 [95% CI 2.84–60.78]; *p* = .001) during a 24‐month FU. As additional findings, serum OPN correlates with intraplaque count of neutrophils, total macrophages and MMP‐9 content.
Yu et al.^147^	2019	82 ACS patients from 4 independent Chinese 2y FU	ACS	OPN increases in ACS patients as compared with healthy controls with an OR_adj_ of 4.64 (95% CI 3.16–6.79). OPN is a predictor of recurrent ACS in a nested case–control sudy OR_adj_ 1.29 (95% CI 1.06–1.57).
Kwee et al.^148^	2019	745 NSTE‐ACS patients from the TRILOGY‐ABSS trial with recurrent (*n* = 108) or not (637) event 1y FU	CV death MI IS	OPN and NT‐proBNP cluster together for the higher fold‐change in NSTE‐ACS‐related miRNA and were significantly associated with the time to first recurrent event with HR_adj_ of 1.20 (95% CI 1.13–1.26) and HR 1.68 (95% CI 1.22–2.32), respectively.
Mohebi et al.^140^	2023	927 CKD patients from CASABLANCA study 2y FU	MACEs	Machine learning algorithm and targeted proteomics allow to identify MACE predictive model including OPN – alongside with KIM‐1, NT‐proBNP, OPN and TIMP‐1 with an HR of 2.82 (95% CI 1.53–5.22) for CKD stage I–II and 8.32 (95% CI 1.12–61.76) for CKD stage III–IV.
Cheong et al.^149^	2023	666 subjects from the Biosignature Registry ≥72‐month FU	All‐cause death AMI	The increased of OPN levels predict worse outcome in terms of all‐cause and CV deaths (HR 1.9 [95% CI 1.29–2.80] and 1.92 [95% CI 1.04–3.55], respectively). However, only the risk of AMI‐related hospitalization is predicted by OPN in adjusted analysis (HR_adj_ 2.22 [95% CI 1.27–3.89])
D'Arrigo et al.^13^	2023	726 CKD patients from MAURO study 3y FU	All‐cause death Non‐fatal CV event	In survival analysis, 1‐unit increase of logOPN levels over time strongly associates with the combined endpoint (HR_adj_ 2.0 [95% CI 1.1–3.6]; *p* = .021) alongside with repeated measurements of CRP.

*Note*: Pubmed search: cardiovascular AND ((risk [Ti]) OR (event [Ti]) OR (death [Ti])) AND ((osteopontin) OR (OPN) OR (SPP1)) and human.

Abbreviations: AAA, abdominal aortic aneurysm; ACS, acute coronary syndrome; AMI, acute myocardial infarction; CAD, coronary artery disease; CEA, carotid endarterectomy; CI, confidence interval; CKD, chronic kidney disease; CRP, C‐reactive protein; CV, cardiovascular; DM, diabetes mellitus; FU, follow up; HF, heart failure; HR, hazard ratio; IS, ischemic stroke; KIM, kidney injury molecule; MACE, major adverse cardiovascular event; MI, myocardial infarction; MMP, matrix metalloproteinase; NSTE, non‐ST elevation; NT‐proBNP, N‐terminal pro B‐type natriuretic peptide; OPN, osteopontin; OR, odds ratio; TIMP, tissue inhibitor of metalloproteinase; u/pOPN, urinary/plasma.

When included in multipanel assays, OPN has an intriguing – but not yet proven – potential to unveil accelerated senescence patterns that may overcome the classical risk factor burden.^5^, ^73^, ^150^ Indeed, OPN appears to capture biological domains – visceral adiposity, metabolic dysfunction, senescence‐related inflammation and early myocardial remodelling – that are not fully encompassed by conventional risk scores. Furthermore, the increasing availability of single‐cell RNA‐sequencing and the advance in statistical analyses now allow the design of an omics atlas for different diseases, including CV ones.^151^, ^152^, ^153^, ^154^, ^155^ Combining the application of artificial intelligence with biomarkers is a future direction with only preliminary available data.^156^

## 6 CONCLUSION AND PERSPECTIVE

Cardiometabolic diseases affect a significant portion of the global population and represent a major public health issue; thus, it is of fundamental importance for healthcare systems to provide adequate assistance (regarding access to medications and services) and, where possible, to assess risk and improve disease prevention.^157^, ^158^ In this regard, several tools have been identified to stratify the risk of these conditions. Risk assessment, in fact, allows for the implementation of early and personalized prevention strategies.^157^ Among these tools, we must mention the focus of our review, OPN, which, as previously mentioned, could play a role as a biomarker for cardiometabolic risk stratification, although it has the limitation of being a pleiotropic marker with low specificity, as it is involved in various conditions and not only in CV ones.

As the so‐called ‘obesity paradox’ largely reflects the limitations of BMI as a crude descriptor of cardiometabolic risk, it is worth noting that OPN may help reconcile this apparent contradiction. Although direct evidence linking OPN to the obesity paradox is still lacking, it is increasingly evident that OPN rises selectively with visceral and ectopic fat accumulation, insulin resistance and senescence‐related inflammation rather than with total adiposity. Consequently, higher OPN levels characterize the transition from metabolically healthy to unhealthy obesity and mirror biological aging more accurately than BMI. This framework supports the view that OPN can unmask hidden risk in patients who appear metabolically preserved despite elevated body weight, thereby offering a biologically grounded perspective on the obesity paradox. To date, OPN has not yet been shown to improve established risk scores, but it clearly has the potential to enrich them by capturing VAT‐driven inflammation and cardiac remodelling. This may be particularly useful in patients with obesity, metabolic dysregulation or discordant BMI–risk profiles, where conventional scores have demonstrated limited sensitivity.

Machine learning (ML) is increasingly playing a prominent role in risk stratification. Atehortúa et al. in their study demonstrated the potential of ML in identifying patients at high risk of CV diseases and type 2 diabetes mellitus using a novel approach that surpasses the limitations of previous studies in the same field. Unlike previous studies that considered a very limited number of exposures, this approach uses a machine learning model based on exposomal factors; an individual's lifetime exposures (exposome) combined with their genome significantly contribute to disease risk. The exposure data processed by ML comes from questionnaires, sensors located in smartphones, computers and wearable electronic devices, which, compared to biological data, are always available and easily accessible to the population.^157^ Another study conducted by Huang and colleagues utilized ML combined with OCTA in the same area of prevention: specifically, to identify the association between cardio‐metabolic risk factors and retinal microvascular alterations in the context of diabetic retinopathy.^159^

Digital health technologies and machine‐learning approaches (including electronic decision‐support tools, telemonitoring, remote monitoring and mobile health applications) have the potential to enhance individualized cardiovascular risk assessment by integrating multiple biological domains—including senescence‐related biomarkers such as OPN—with clinical, imaging and exposomic data. Although still at an early stage of development, these tools may ultimately enable more refined stratification of patients whose residual cardiometabolic risk is not fully captured by traditional parameters. In this context, AI‐driven platforms should be viewed as complementary to biomarker‐based approaches rather than standalone strategies. Such technologies may also support healthcare systems in achieving broader and more equitable access to cardiovascular prevention and care, thereby reducing CVD‐related mortality.^157^, ^158^ Such technologies may also support healthcare systems in achieving broader and more equitable access to cardiovascular prevention and care, thereby reducing CVD‐related mortality.^158^

## CONFLICT OF INTEREST STATEMENT

Luca Liberale is co‐inventor on the international patent WO/2020/226993 filed in April 2020. The patent relates to the use of antibodies which specifically bind IL‐1α to reduce various sequelae of ischemia–reperfusion injury to the central nervous system. Luca Liberale reports speaker fees from Daiichi‐Sankyo outside the submitted work and has received funding from the Novartis Foundation for Medical‐biological Research (unrelated to this work). The other authors declare they have no conflict of interest.

## Data Availability

Not applicable.
